# Detection of atrial fibrillation with implantable loop recorders in horses

**DOI:** 10.1111/evj.13301

**Published:** 2020-06-28

**Authors:** Rikke Buhl, Eva M. Hesselkilde, Helena Carstensen, Merle F. Fenner, Thomas Jespersen, Jacob Tfelt‐Hansen, Stefan Michael Sattler

**Affiliations:** ^1^ Department of Veterinary Clinical Sciences Faculty of Health and Medical Sciences University of Copenhagen Copenhagen Denmark; ^2^ Department of Biomedical Sciences Faculty of Health and Medical Sciences University of Copenhagen Copenhagen Denmark; ^3^ Department of Cardiology The Heart Centre Copenhagen University Hospital Copenhagen Denmark; ^4^ Department of Forensic Medicine Faculty of Medical Sciences University of Copenhagen Copenhagen Denmark

**Keywords:** horse, cardiac arrhythmia, atrial fibrillation, implantable loop recorder, cardiology

## Abstract

**Background:**

Cardiac arrhythmias in horses are diagnosed by auscultation or electrocardiogram (ECG), which results in a low sensitivity for detecting arrhythmias that occur sporadically. Implantable loop recorders (ILRs) are small ECG devices placed subcutaneously, to automatically detect arrhythmias in human patients.

**Objectives:**

To test ILRs ability to detect atrial fibrillation (AF) in horses. Furthermore, we hypothesised that anatomical location of the implant site might influence signal quality. Signal quality was evaluated both during exercise and over time.

**Study design:**

Experimental study.

**Methods:**

In five Standardbred mares, eleven ILRs were implanted subcutaneously in up to three different positions (Front: pectoral region, Left‐6: sixth left intercostal space and Ventral: xiphoid region) and AF induced. The R‐ and T‐wave amplitudes were measured in all positions over time during AF. AF burden automatically registered by the ILRs over a 2‐month period was compared with selected Holter ECG recordings.

**Results:**

All three positions had stable R‐ and T‐wave amplitudes during the study period and were of sufficient quality to allow AF detection at rest. The position Left‐6 showed significantly higher R‐ and T‐wave amplitudes compared with the other positions. During submaximal exercise only the Left‐6 position was able to record ECG signals of diagnostic quality. No position yielded diagnostic signals at maximum exercise due to artefacts.

**Main limitations:**

Few horses and ILRs included and no spontaneous AF episodes were studied.

**Conclusions:**

This preliminary study indicates that ILRs can be used for AF detection in horses, but the anatomical location is important for optimal ECG quality. Despite insufficient quality during exercise, ILRs were suitable for AF detection at rest. Therefore, the ILR may be a valuable diagnostic tool for detecting paroxysmal AF in horses.

## 1 INTRODUCTION

Cardiac arrhythmias occur relatively often in horses both during rest and exercise. Although most arrhythmias are without clinical significance,^1^, ^2^ atrial fibrillation (AF) is a common pathological arrhythmia affecting performance in horses.^2^, ^3^, ^4^, ^5^, ^6^

Diagnosis of AF is based on cardiac auscultation, confirmed by an electrocardiogram (ECG) showing irregular RR intervals with normal QRS complexes, absence of P waves and the presence of f waves. A Holter ECG may be obtained in cases where paroxysmal AF (PAF) is suspected. Also heart rate monitors are able to distinguish AF from sinus rhythm (SR).^7^ However, for practical reasons, the duration of heart rate monitor recordings or Holter ECGs is limited to a few days and electrodes can be cumbersome to keep in place. As a result, short‐lasting AF episodes may not be diagnosed. This is particularly problematic as long‐lasting AF becomes more resistant to treatment, and early recognition of the disease is therefore essential.^8^ Interestingly, a recent study has shown changed treatment strategy in people with PAF diagnosed with ILRs to prevent chronic AF and co‐morbidities.^9^

Implantable loop recorders (ILRs) can overcome time limitations of Holter ECGs. First applied almost 20 years ago in human patients experiencing unexplained syncope,^10^ now their application has been extended to detect AF.^11^, ^12^ The ILRs are subcutaneously implanted devices that continuously analyse an ECG of the patient. Based on changes in RR interval, ILRs are able to automatically detect predefined arrhythmic events such as bradycardia, tachycardia, AF and asystole. Unlike Holter recordings, no continuous ECG is stored, rather clinicians are provided with a report on detected arrhythmias including short ECG examples. In humans, ILRs are used to detect PAF or AF recurrence in asymptomatic patients, with greater diagnostic yield than conventional Holter ECG.^11^, ^12^, ^13^, ^14^, ^15^ In veterinary medicine, ILRs have been used in dogs with unexplained syncope in order to identify cardiac causes,^16^, ^17^, ^18^ and in three horses to exclude cardiac arrhythmias as the triggering cause of collapse.^19^

The aim of the current study was to test ILRs ability to detect AF in horses. We hypothesised that anatomical location of the implant site might influence signal quality. Furthermore, signal quality was evaluated both during exercise and over time.

## 2 MATERIALS AND METHODS

### 2.1 Experimental protocol

**Figure 1:**
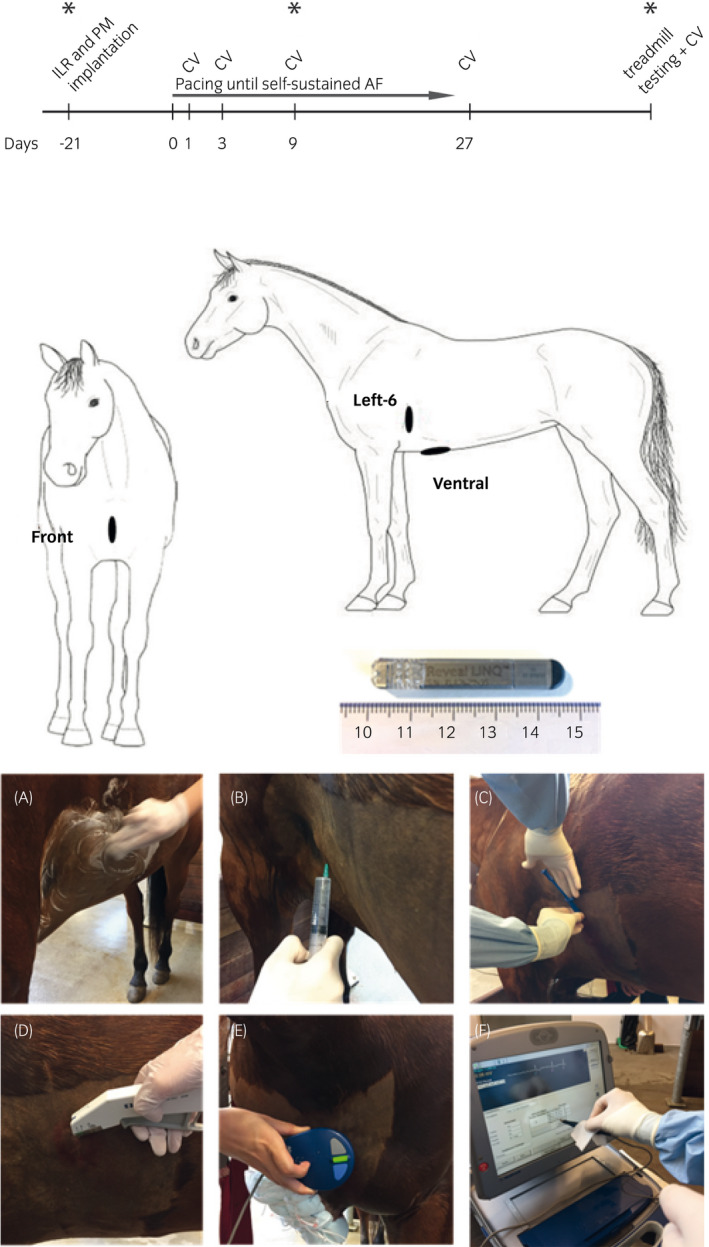
Schematic illustration of timeline of the study (above), anatomical position of the three implantable loop recorders (middle) and implantation procedure (below). ILR, implantable loop recorder; PM, pacemaker implantation; CV, cardioversion. *Interrogation of the ILR, “Front”: Superficial pectoral region, “Left‐6”: The sixth left intercostal space at the level of the shoulder joint, “Ventral”: The level of the xiphoid process in a cranio‐caudal direction. Picture of the implantable loop recorder used (Medtronic reveal LINQ). Implantation sequence (A‐D) and interrogation with the programmer (E, F) are shown

Five Standardbred mares with a mean age of 8.6 years (range 5‐13 years) and a mean bodyweight of 471 kg (range 320‐572 kg) were included. This study used a subset of horses that were used to evaluate the longitudinal effect of AF in horses (timeline, Figure 1) and the horses were subjected to euthanasia and post‐mortem examination at the end of the study. Atrial fibrillation was induced by tachypacing the right atrium with pacemakers implanted in local anaesthesia and leads were placed in the right atrium through the right cephalic vein as previously described.^20^

The Medtronic Reveal Linq ILRs (Medtronic Inc.) (size 44.8 × 7.2 × 4.0 mm, weight 2.5 g) were implanted at different anatomical positions along with pacemaker implantation (Figure 1). Skin was clipped and surgically prepared, and 10 mL local analgesia was applied subcutaneously (Lidocain, Xylocaine^®^, AstraZeneca). ILRs were inserted through a 1 cm incision into a subcutaneous pocket, anchoring the device to the muscular plane. They were kept in place by the surrounding tissue without further fixation. Skin was stapled using two skin staples (Appose VLC Auto suture, Coviden) and covered with sterile adhesive dressing (Sorbact, ABIGO Medical). The operation (from incision to closure) lasted less than 5 minutes. The horses were treated with 1 mg/kg bodyweight (bwt) flunixin intravenously (Finadyne^®^, MSD Animal Health) for 3 days.

Implant sites were chosen after evaluating surface ECGs (KRUTECH Televet, Kruuse A/S) on different locations. Furthermore, the positions should not interfere with saddle or girth. In total, 11 ILRs were implanted in five horses. Three horses had three ILRs implanted subcutaneously: one in the superficial pectoral region (Front), one at the sixth left intercostal space at the level of the shoulder joint approximately 15 cm above the level of the left olecranon (Left‐6) and one ventrally at the level of the xiphoid process (Ventral) (Figure 1), and two horses had one ILR implanted at Left‐6. Following implantation of pacemaker and ILRs, the horses had a resting period of 3 weeks where the performance of ILRs in SR could be evaluated. Hereafter, the horses were paced into sustained AF. As the horses were part of two pharmacological studies, cardioversion was attempted on days 1, 3, 9 and 27 following AF induction with intravenous infusion of 2 mg/kg flecainide (Tambocor^®^, Meda AS) or 3 mg/kg Acetylcholine‐Activated Inward‐Rectifier K^+^ Current blocker (XAF‐1407, Xention Ltd.). AF was re‐induced after successful cardioversion.

Four horses underwent an incremental treadmill testing to fatigue when in persistent AF as previously described.^6^ During exercise, ILRs were manually activated (a 10‐minute ECG trace can be recorded after manual activation) to cover the latest part of the warm‐up period, the exercise test to fatigue and immediate post‐exercise period. The horses were equipped with a head collar and an elastic girth to attach the surface ECG.

### 2.2 Implantable loop recorder

**Figure 2:**
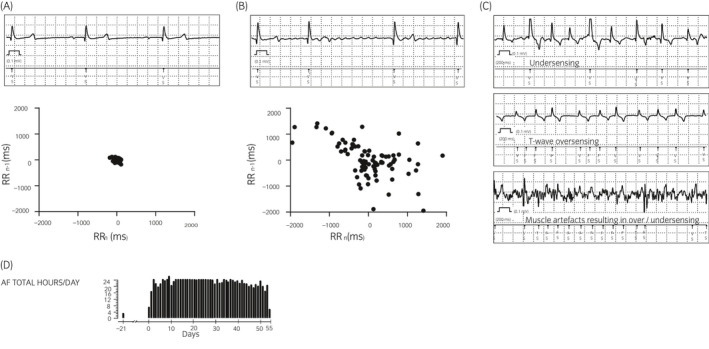
Examples of electrocardiograms (ECGs) and Lorenz plots at rest in sinus rhythm (A) and atrial fibrillation (B) recorded by the implantable loop at location Left‐6. Artefact recordings (C) corresponding to undersensing, T‐wave oversensing and muscle artefact over/undersensing. Representative AF burden histogram (D) showing h/d of AF registered by a single ILR

Implantable loop recorders continuously obtain ECG signals to detect RR intervals. Detection of arrhythmia (bradycardia, tachycardia, AF and asystole) is based on these RR intervals. The AF detection algorithm uses a 2‐minute ECG interval to detect variation in the RR intervals. The variation is measured by plotting the current RR interval vs the RR interval of the preceding heart beat in a Lorenz Plot (Figure 2). To refine AF detection, additionally the ILR searches for a P wave between two R waves. A list containing all types of arrhythmia and its respective number of occurrence is stored automatically for the total lifetime of the device. Additionally, the reveal LINQ can store a list containing up to 30 episodes in greater detail, including date and time of onset, duration as well as mean and maximal heart rate. This list is complemented with up to 14 ECG traces (examples are shown in Figure 2). The ECGs carry annotations under each detected R wave and can be used to retrace the ILRs diagnose. If more episodes occur, the ECG of the oldest episodes will be overwritten, but the list containing the total count will not be affected by that. Total duration of AF episodes is stored as AF burden (percentage in AF per day). Recorded episodes can be downloaded using a programming device or transferred automatically to Medtronic's care link server every 24 hours using a home monitoring device. Battery life is typically 3 years depending on the model. For further details on the functionality of the ILR, the manufacturer's manual can be consulted.

In this study, each ILR was interrogated immediately after insertion using the programming device (2090 Medtronic, Reveal Linq, Medtronic Inc.) when the horses were still in sinus rhythm (SR). To perform an interrogation, the programming head was placed directly on the skin over the implanted ILR and kept in position to establish a connection (Figure 1). When initialising the ILR, the following were chosen by the predefined standard settings; sensing threshold (defines the minimum height of a signal to be detected as a heartbeat [R wave]) = 0.035 mV and blanking after sense (defines the minimal time before a new R wave can be detected) = 150 ms. Also, the detection for arrhythmia of interest (AF, tachycardia and pauses) have to be turned on. For this study we only focused on automatic detection of AF (threshold ≥6 minutes). If manually activated, the ILRs were set to record 10 minutes of ECG (9 minutes before and 1 minute after the manual activation).

### 2.3 Data collection

Interrogations with data transfer from the ILR were performed 1 month (range 28‐35 days) and 2 months (range 52‐85 days) after implantation. During each interrogation, a protocol including a list with the last 30 episodes as well as AF burden measured as hours/d since device implantation was exported as a PDF file to a memory stick. After the interrogation all data stored were automatically removed from the ILR and new episodes could be stored.

Holter ECG recordings (KRUTECH Televet, Kruuse A/S) were obtained before AF induction and various times during AF and analysed manually to verify correct AF detection of the ILR. Holter ECGs were recorded on average for 14.4 days (range 13‐16 days), resulting in ECGs of diagnostic quality from the Holter recordings of a mean duration of 145 hours (range 106‐288 hours) for each horse.

### 2.4 Data analysis

Exported PDF report files were used for offline analysis using PDF‐X Change (Version 2.5, Tracker Software Products Ltd.). The AF burden (AF hours/d) was extracted from the AF burden histogram and two physicians reviewed all episodes that were classified as AF using ECG examples from the ILRs to ensure correct detection. ECG recordings were available at 25 mm/s sweep speed with a calibration pulse at the start of each recording. PDF‐X change was used to perform calibration while measuring the ECG recordings.

As the ILR algorithm relies on R‐wave detection, the amplitude of the R and T waves was measured from 10 consecutive heart beats from registered AF episodes obtained approximately 1 and 2 months after ILR implantation. The average of R‐ and T‐wave amplitude as well as the presence of f waves were compared for all the three locations. To evaluate the ECG over time, the R‐ and T‐wave amplitude was compared between first and second interrogation.

A 2‐way ANOVA test was used to compare amplitude of R and T waves (position and time) (GraphPad Prism, Version 7.02, GraphPad Software Inc.). *P* < .05 was considered statistically significant. All values are represented as mean ± SD if not stated otherwise.

## 3 RESULTS

All 11 ILRs were successfully implanted without complication. However, during the interrogation after 1 month, one ILR at ventral position was defective and no data were recorded. Therefore, only data from two ILRs at the Ventral position were analysed. In total, 10 ILRs could be used for analysis and mean duration of ILR time per horse from insertion until the last interrogation of the ILR was 70 days (range 52‐85 days).

### 3.1 Anatomical location and signal quality at rest

The R‐ and T‐wave amplitudes for the three ILR positions are shown in Table 1. The R waves were positive in all three positions and significantly higher in the Left‐6 position (0.52 ± 0.03 mV; for an example, see Figure 1), compared with the Front and Ventral positions (0.12 ± 0.01 and 0.13 ± 0.01 mV, respectively; *P* < .0001). The T wave was positive in Left‐6 and negative in front and ventral position. Its amplitude was significantly higher for the Left‐6 (0.15 ± 0.01 mV) compared with the Front and Ventral positions (0.03 ± 0.01 and 0.06 ± 0.02 mV, respectively; *P* < .0001). The R/T amplitude ratio was 3.5, 4.0 and 2.2 for Left‐6, Front and Ventral positions respectively. The f waves were visible in all recorded AF episodes for the Left‐6 position, and to a lesser degree for the Front (67%) and Ventral positions (0%; Table 1). Amplitudes remained stable over time since no significant differences between R‐ and T‐wave amplitude were observed between the measurements obtained approximately 1 and 2 months after implantation (Table 1). ILRs were removed post‐mortem. The connective tissue in contact with ILRs showed mild calcification. Otherwise, no signs of inflammation could be observed.

**Table 1:** Mean ± SD of R‐ and T‐wave amplitudes from 10 consecutive beats and the percentage of QRS complexes with preceding f waves during AF for the three implantable loop recorders approximately 1 and 2 mo after implantation

	Left‐6 ILR (N = 5)			Front ILR (N = 3)			Ventral ILR (N = 2)		
	R (mV)	T (mV)	f waves	R (mV)	T (mV)	f waves	R (mV)	T (mV)	f waves
1 mo	0.49 ± 0.07	0.14 ± 0.06	100%	0.12 ± 0.02	0.03 ± 0.02	73%	0.13 ± 0.03	0.04 ± 0.02	0%
2 mo	0.54 ± 0.06	0.15± 0.05	100%	0.11 ± 0.02	0.02 ± 0.02	60%	0.13 ± 0.03	0.07 ± 0.1	0%

Note

f waves: percentage of 10 consecutive QRS complexes that showed preceding f waves.

Abbreviation: ILR, implantable loop recorder.

### 3.2 Signal quality during exercise

In the four horses tested on the treadmill, the ILRs sensed R waves correctly in the triggered ECG recordings for all positions at rest and during walking. During submaximal exercise (warm‐up trot at 5 m/s) and immediately after cessation of the maximum exercise test, the quality was only sufficient in the Left‐6 position, while the Front and Ventral positions showed poor diagnostic quality during submaximal exercise. No position yielded diagnostic signals at maximum velocity due to artefacts. When the horses were walking at the treadmill immediately after the test, the quality was sufficient for the Left‐6 position and regained sufficient quality for the other positions 1‐2 minutes after the test was stopped.

### 3.3 Atrial fibrillation burden

AF burden showed similar durations for the different ILR positions in the individual horses. An example of AF burden is given in Figure 2. Overall, all ILRs could detect AF episodes on days where the horses were in AF (diagnosed by auscultation or Holter ECG). The AF burden registered at different location differed slightly.

All horses had been in sinus rhythm for 3 weeks with ILRs implanted (day −21 to 0). None of the ILRs detected a false‐positive AF episode during this period. However, horses were kept in box rest the first 2 weeks and allowed paddock turn out in week 3.

### 3.4 Artefacts and heartbeat misclassification

Undersensing and/or oversensing of R waves (Figure 2) in ECG traces recorded with detected AF were rare. Undersensing was typically observed when intermittent beats with lower R‐wave amplitude were present. Further classification of these beats was not possible from the recorded ECG traces. Oversensing occurred when the R/T‐wave amplitude ratio was low. This was typically the case for T waves with high amplitude that were misclassified as R waves, regardless whether the T wave was positive or negative.

In individual cases, episodes of 10‐20 seconds lasting artefacts due to movement or muscular activity led to intermittent over‐ and undersensing of R waves (Figure 2). Since this phenomenon was short lasting, it did not lead to the detection of false‐positive AF episodes. Overall, the Front and Ventral ILR were most prone to muscle artefacts.

## 4 DISCUSSION

This study showed that ILR implantation in horses is feasible and safe and that the obtained ECG amplitudes are stable over time. It is possible to detect experimentally induced AF, but the anatomical position is crucial for a high‐quality ECG recording and correct detection. Movement artefacts increased during exercise and thereby reduced the diagnostic quality of the recordings.

The ILR’s arrhythmia detection algorithms are R‐wave dependent, therefore, high R‐wave amplitudes (to ensure a good signal‐to‐noise ratio) and a high R/T amplitude ratio are essential to correctly identify arrhythmias. The left lateral position (Left‐6) was superior to the other positions used in the present study. At this location the ILR could potentially interfere with the saddle girth, although in most horses the girth would be located caudal to the position. The only previous reported study in horses using ILR implanted the ILR at the front pectoral region to detect cardiac arrhythmias as an explanation for collapse. The ILR used in the previous study did not detect arrhythmias during the two recorded episodes of collapse.^19^ Our study suggests that this position is suboptimal as the Front position was associated with low R‐wave amplitude and a high prevalence of artefacts.

The quality of the ECG recordings from the ILRs remained stable over time, which support the anticipation that these recorders are capable of recording sufficient quality ECGs over longer time in horses as it is seen in humans.^21^ However, artefacts were seen and could potentially lead to false‐positive registration of AF. ILR can therefore not replace the manual analysis of a Holter ECG, which is the Golden Standard for AF detection, but the ILR could be useful in suspected cases of intermittent AF as they provide continuous arrhythmia detection over long time.

The performance of ILRs during exercise showed artefacts due to myopotential interference resulting in poor‐quality ECGs for most positions in the present study. Only the Left‐6 position showed acceptable ECGs during trot at 5 m/s. Studies in humans using ILRs in a setting where exercise has been performed have functioned sufficiently,^21^, ^22^, ^23^ although it is not described in details. One study that investigated effect of training on AF burden reported up to 2.7% false‐positive AF episodes due to artefacts during activity, ectopic beats or sinus arrhythmia.^22^ These results are in accordance with our findings where none of the detected AF episodes documented with ECG by the ILR were false positive due to artefacts.

The Reveal LINQ is equipped with a built‐in algorithm that refrains noisy signals from being used in arrhythmia detection. The intervention of this algorithm or the amount of rejected signals is not presented to the clinician and a quantitative analysis is therefore not possible. However, the ILR rejected ECG episodes superimposed with artefacts (ie recordings during maximal exercise) and did not use them to detect an arrhythmia‐like AF. Despite this limitation, the AF burden histogram for the specific days showed that AF occurred for almost 24 hours/d for the horses, which indicate that the automatic artefact reduction is low in horses at rest. Furthermore, we did not observe false‐positive AF episodes during sinus rhythm, before AF induction pacing was turned on.

A limitation of the study was the low number of horses included and that not all horses had three ILRs implanted, which should be taken into consideration when interpreting the data. Furthermore, interrogation was only performed on three defined time points and more examples of ECG recordings could have been exported and analysed if interrogations were performed on a weekly basis. However, the episode lists were never full when the ILRs were interrogated and therefore no AF episodes were deleted by the ILR before interrogation. The AF episodes in the present study were all induced by tachypacing and therefore the diagnostic capability of the ILRs in horses developing spontaneous AF needs further exploration. Also, it is relevant to study horses over a longer time period whether false‐positive AF episodes would be registered in horses in SR, ie due to marked sinus arrhythmia.

## 5 CONCLUSIONS

With this method, detection of AF at rest and during moderate exercise in horses is possible, especially for the Left‐6 position. Furthermore, a detailed understanding of the relationship among clinical signs of AF, AF initiation and duration may be obtained in future studies. As a supplement to conventional Holter ECG, ILRs are likely to improve the diagnostic routines for AF detection, provide researchers and clinicians with a better knowledge of the actual AF burden, AF recurrence rate, prevalence of PAF and an understanding of the triggering factors of AF in horses. For maximum exercise tests, surface ECGs remain the best diagnostic option.

## CONFLICT OF INTERESTS

No competing interests have been declared.

## AUTHOR CONTRIBUTIONS

R. Buhl designed the study, analysed the data and drafted the manuscript. E. Hesselkilde designed the study, collected the data and analysed the data. H. Carstensen designed the study and collected the data. M. Fenner collected the data and analysed the data. T. Jespersen was a major contributor writing the manuscript. J. Tfelt‐Hansen assisted in designing the study and performing the surgery. S. Sattler designed the study, analysed the data and drafted the manuscript. All authors read and approved the final version of the manuscript.

## Ethical animal research

The Danish Animal Experiments Inspectorate (2015‐15‐0201‐00693) as well as the local ethical committee at the Department of Veterinary Clinical Sciences, University of Copenhagen, approved the study.

## Owner informed consent

Written owner‐informed consent was obtained prior to the study.

## Data Accessibility Statement

The data are available from the corresponding author on reasonable request.
